# Research on emotion animation generation based on AIGC tools: Personalized expression and multi-level encoding strategy

**DOI:** 10.1371/journal.pone.0356895

**Published:** 2026-08-25

**Authors:** Yitian Liu, Zhou Zhao, Kun Qi

**Affiliations:** 1 School of Media Arts and Communication, Nanjing University of the Arts, Nanjing Jiangsu, China; 2 College of Communication, Nanjing University of the Arts, Nanjing, Jiangsu, China; 3 School of Electronic and Communication Engineering, Shenzhen Polytechnic University, Shenzhen, Guangdong, China; University of Messina, ITALY

## Abstract

This study targets the problems of poor semantic consistency and inadequate control over personalized expression in emotion animation generation. Its goal is to develop an automated generation framework that combines multi‑level semantic recognition with style adjustment functions, thereby enhancing the semantic building system and human‑computer interaction paradigms of AIGC technology for controllable emotional animation. Utilizing ChatGPT’s NLP capabilities and Runway ML’s techniques for generating dynamic images, we construct a hierarchical prompt structure and adopt a three‑dimensional encoding strategy, which allows structured interpretation and coordinated mapping of emotional, behavioral, and stylistic information. The experimental results demonstrated that the proposed method could stably generate animations with coordinated consistency among facial expressions, motion rhythm, and color semantics under various emotion-driven conditions, while exhibiting reasonable temporal progression characteristics. In personalized generation, distinct user profiles produced significant differences in dimensions such as expression amplitude, motion rhythm, and stylistic features, reflecting the model’s fine-grained control over multidimensional generation parameters. In the validation of the encoding mechanism, the integration of hierarchical prompts with the multi-level encoding strategy significantly enhanced the naturalness of facial expressions, the logical coherence of movements, the continuity of temporal sequencing, and the precision of micro-action depiction, thereby reinforcing the intensity and realism of emotional presentation. This work develops a framework for generating emotional animations that combines semantic layering with adaptive personalization. It offers methodological backing for the structural optimization and interactive enhancement of AIGC systems within the realm of high‑quality visual content production.

## 1. Introduction

The growing convergence of digital technology with affective computing has led to the broad application of emotional animation—a visually expressive medium primarily guided by emotional states—across areas like virtual companionship, digital therapy, creative industries, and educational communication [1,2]. Unlike traditional narrative‑based animation, emotional animation prioritizes subjective experiences and emotional exchange, and its generation process often depends on the integrated modeling of multiple emotional dimensions such as language, facial cues, and body movement [3]. Thus, precisely identifying user emotions and enabling personalized expression within animated content have become key drivers for making emotion‑driven animation generation more intelligent.

Artificial Intelligence Generated Content (AIGC) refers to a technical framework that employs generative AI models to automate content creation across multimodal domains, including text, images, audio, and video [4]. Unlike traditional rule‑based or template‑driven approaches, AIGC leverages deep neural networks to deliver robust semantic comprehension, style transfer, and multimodal fusion capabilities, allowing for the generation of creative and adaptable content based on user input [5]. The expanding application of AI‑generation tools in animation is driving a transformation from manual drawing to intelligent synthesis and from linear modeling to cross‑modal collaboration. In the initial stages of content conceptualization and scriptwriting, language generation models are commonly deployed to extract emotional semantics, construct character psychology, and automatically produce scene‑setting texts, thereby greatly enhancing both semantic consistency and script generation efficiency in emotion‑driven animation [6,7]. For visual content creation, platforms such as Runway ML, Pika Labs, and Kaiber allow users to quickly generate animated clips featuring dynamic elements and stylistic variations by using structured prompts, thereby providing efficient technical pathways for the visualization of emotional content [8]. Additionally, in the areas of animation sequencing and style control, certain tools have integrated functionalities for keyframe editing, expression‑based adjustment, and temporal management, achieving preliminary controlled mapping from emotional descriptions to dynamic visual outputs and expanding AI’s expressive capabilities in animation synthesis [9]. In recent years, the rapid advancement of diffusion models and text-to-video generation techniques has significantly accelerated the application of AIGC in the field of visual content generation [10–12]. Representative text-to-video models, such as Sora, have demonstrated strong capabilities in temporal modeling and scene consistency, enabling more coherent and contextually aligned video synthesis [13,14]. Meanwhile, models such as Pika have continuously improved in motion generation and dynamic control, progressively enhancing the operability and expressive potential of animation generation driven by natural language inputs [15]. Current AIGC tools for animation generation, however, remain largely focused on generic features like scene rendering, motion generation, and contextual building, with the primary research emphasis placed on boosting generation efficiency and visual quality. A structured solution that is specifically adapted to emotion‑dominant generation tasks has yet to emerge [16,17]. In response to these challenges, this paper proposes an AI‑driven emotional animation generation approach that establishes a three‑stage closed‑loop framework—“emotion recognition → prompt generation → animation output”—aimed at achieving precise semantic modeling of emotions and personalized expression of dynamic visual content. The results demonstrate the practicality and effectiveness of the collaborative interplay between language generation tools and dynamic image platforms within emotion‑driven animation, thus delivering improved emotion‑aware generation methods for human‑computer interaction, psychological expression, and immersive storytelling applications.

The paper is structured as follows. The first section covers the research background, current developments, and the relevance of this work. The second section systematically surveys the progress made in the field of emotional animation generation. The third section introduces the proposed overall framework, including mechanisms for emotion recognition, strategies for prompt design, methods for personalization modeling, and the logic behind constructing a multi‑level encoding structure. The fourth section illustrates animation generation results by analyzing typical emotion cases and variations in user profiles, and assesses both expression accuracy and personalized adaptation capabilities. The fifth section provides a thorough analysis of the theoretical contributions of the results and points out limitations. The sixth section summarizes the conclusions and proposes avenues for advancing future research.

## 2. Literature review

Emotion-driven animation generation integrates affective computing with visual expression, and its research foundation is primarily rooted in emotional psychology and animation principles [18],Cai, 2024). With the continued development of affective computing, the recognition and structured representation of emotional semantics have gradually become a critical foundation for intelligent animation generation. Existing studies can generally be organized from three perspectives: emotion modeling, animation generation technologies, and prompt engineering. The first focuses on the semantic representation of emotional states, whereas the latter two are concerned with visual generation mechanisms and control strategies under natural language guidance, respectively. A review conducted within this analytical framework helps clarify the technical pathways and evolutionary characteristics of emotion animation generation.

In the domain of emotion modeling, relevant studies commonly rely on dimensional emotion theories or basic emotion classification systems to achieve structured representations of emotional states, thereby providing animation systems with recognizable input foundations ([19,40]. By transforming subjective emotions into standardized semantic units, such approaches enable systems to determine and constrain emotional states prior to generation. As research has progressed, emotion modeling has gradually shifted from static label recognition toward the characterization of emotional intensity, temporal variation, and the relationship between emotion and behavioral expression, allowing emotional information to exert a stronger regulatory role in the animation generation process. Overall, this line of research establishes the necessary semantic basis for emotion-driven animation and provides the prerequisite for the subsequent organization of visual expression.

With respect to animation generation technologies, early studies primarily relied on rule-driven and template-matching mechanisms, in which emotion labels were mapped onto predefined expression or motion libraries to enable animated characters to switch across different emotional states [20]. Although such methods feature clear structural logic, they remain limited in expressive richness and dynamic continuity. To improve generation quality, some studies introduced multimodal inputs by combining speech prosody and textual information for emotion inference, while further enabling coordinated modeling of facial expressions and bodily movements in animation generation [21,22]. To a certain extent, these approaches enhanced the completeness and coherence of emotional expression, allowing animation generation to gradually evolve from single-label-driven mechanisms to multi-source information-driven processes.

On this basis, animation generation research has further expanded toward visual style control and hierarchical semantic organization. Visual elements such as color, lighting, and cinematographic rhythm have been incorporated into the emotional expression process to strengthen the overall perceptual effect. Wang and Sun [23] pointed out that different color tones and compositional strategies can significantly influence emotional perception and may guide generative style through semantic descriptions. Meanwhile, semantic organization has gradually evolved from a single-layer structure to a hierarchical arrangement. Pan et al. [9] and Yan [24] constructed semantic structures integrating emotion, character, and scene in order to improve behavioral logic consistency in animation generation. Kim et al. [25] further incorporated users’ cultural backgrounds and aesthetic preferences as control variables, endowing animation generation with a preliminary capacity for individualized expression. At this stage, the research focus shifted from basic generative capability toward expressive consistency and personalized adaptation.

In the field of prompt engineering, the development of AIGC tools has reshaped the implementation pathway of emotion-driven animation generation. Platforms such as Runway ML, Pika Labs, and Kaiber support animation generation based on natural language, allowing users to directly participate in the generation process through textual descriptions [26,27]. Against this background, prompts have gradually become a key control medium connecting emotional semantics with visual output. Dhamyal et al. [28] showed that emotional vocabulary, behavioral descriptions, and scene information embedded in prompts can be parsed into visual parameters and subsequently influence dimensions such as character movement, expressive intensity, and visual style. Research on prompt structure has continued to deepen. Lv et al. [29] proposed dividing prompts into hierarchical components such as character identity, emotional state, behavioral description, and situational context in order to improve the structural completeness of generation results. Song et al. [30] embedded user characteristics into prompts, making generated content more targeted. Wu et al. [31] further introduced temporally oriented semantics by describing emotional transition processes, thereby guiding the generation of animation sequences with progressive characteristics. These studies indicate that prompts have evolved from simple input forms into control mechanisms with organizational functions. Research on large language models has further demonstrated that models can accomplish task adaptation and output generation under the guidance of prompts and contextual information, suggesting that prompt design itself has become an important factor influencing generation quality and task performance [32]. In this sense, the role of prompts is no longer confined to the input content itself, but has gradually evolved into a methodological carrier that performs organizational, constraining, and guiding functions within generative tasks.

Although emotion-driven animation generation has achieved preliminary progress in terms of expressive dimensions and representational depth, thereby providing the present study with theoretical and technical foundations in emotion modeling, visual element coordination, and prompt-driven generation, existing methods still exhibit several evident limitations. These are mainly reflected in the lack of systematic prompt structure design, the difficulty of ensuring the consistency and stability of generated content, and the fact that personalized control often remains at the level of superficial tag settings without realizing a deep linkage between user characteristics and expressive style. In addition, at the level of emotion modeling, some studies involve both emotion classification models and dimensional models, yet fail to specify a clear theoretical choice and unified mode of representation in practical application, resulting in an insufficiently explicit mapping mechanism of emotional semantics during generation. In response to the requirements of emotion animation generation for semantic controllability and expressive stability, the present study adopts a discrete representation based on basic emotion categories, classifying emotional states into typical categories such as happiness, sadness, anger, and surprise. On this basis, a multi-level encoding strategy is introduced to structurally organize emotion labels, personality traits, and behavioral intentions, thereby enhancing the semantic consistency and expressive precision of animation generation.

## 3. Research design

**Fig 1:**
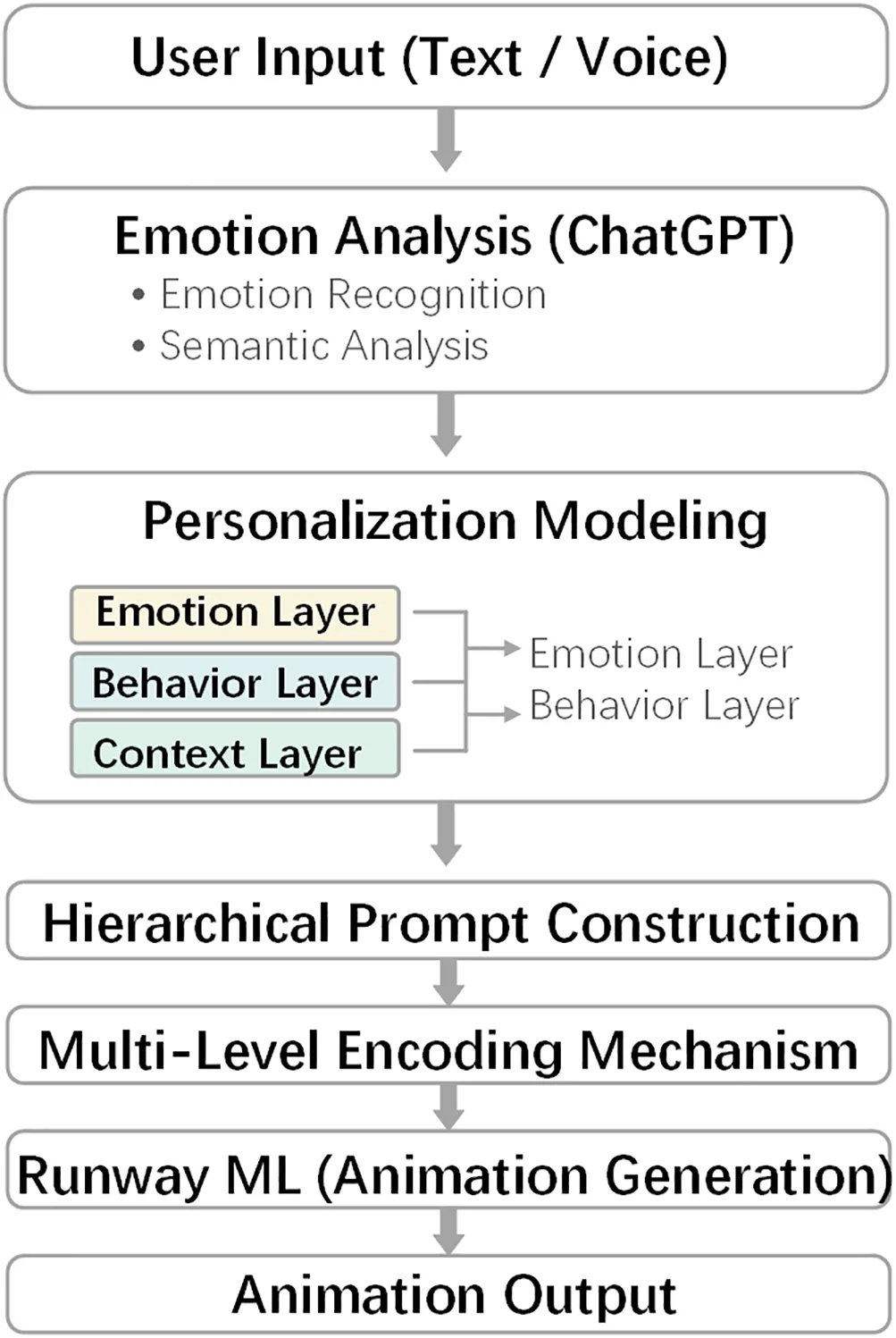
Architecture of the personalized emotion-driven animation generation framework based on AIGC tools.

To systematically present the overall operational mechanism of the proposed method, this study constructs an AIGC-oriented framework for emotion-driven animation generation. As illustrated in Fig 1, the framework takes user input as the starting point and performs semantic recognition through emotion analysis, followed by the integration of user characteristics based on a personalized modeling mechanism. Building upon this, a hierarchical prompt structure is established to organize and encode emotional information, behavioral features, and contextual elements. Through a multi-level encoding mechanism, collaborative mapping across different semantic layers is achieved, ultimately enabling the generation of visual content on the animation platform.

### 3.1. Emotion analysis and prompt design

Within the full pipeline of emotion‑driven animation generation, emotion analysis and prompt design together constitute the essential link that connects content input with visual synthesis. This research utilizes ChatGPT as a language generation tool and Runway ML as a dynamic image generation platform to build a collaborative framework that requires no programming. Through the coordinated operation of these two types of AIGC tools, the entire process from user intent parsing to emotion animation output is realized in an automated manner. This approach not only aligns with a design-oriented workflow but also preserves the core research focus on personalized expression and multi-level emotional encoding.

During the emotion analysis stage, the system leverages ChatGPT’s strong capabilities in semantic understanding and contextual reasoning to perform context-aware processing and emotional semantic inference on user-provided textual or vocal inputs. For textual inputs, the model integrates semantic cues to preliminarily identify the underlying emotion category, textual intensity characteristics, and potential expressive tendencies. For speech inputs, the audio is first transcribed into text, after which emotion label extraction and information structuring are conducted within a unified semantic analysis framework. In terms of emotion modeling, this study adopts a discrete representation framework based on basic emotion categories, drawing on the classification principles of basic emotion theory [33]. Emotional states are categorized into a set of typical classes with clearly defined semantic boundaries, while variations in intensity and expressive tendency are further characterized within each category. The emotion labeling process is neither entirely manually assigned nor fully dependent on automated model outputs; instead, a hybrid strategy is employed, consisting of an initial inference by ChatGPT followed by manual verification and refinement by researchers. The basic emotion categories considered in this study include happiness, anger, sadness, and surprise. Emotional intensity is not treated as an independently predicted continuous variable; rather, it is inferred through a comprehensive assessment of emotional vocabulary, degree modifiers, tonal cues, and subsequent behavioral descriptions present in the input, and is represented using qualitative levels (e.g., low, medium, high) or corresponding intensity descriptors. On this basis, the model further integrates user-defined character attributes—such as age, personality traits, and background information—to construct a concise user profile, thereby providing semantic support for the personalized formulation of prompts.

The prompt design phase serves as the critical bridge between semantic meaning and visual output within the generation pipeline. Aiming for multi‑level encoding and fine‑grained control, this study proposes a layered prompt construction strategy. This approach typically comprises three tiers: 1) clear directives that define emotion categories and intensity levels, laying the groundwork for the animation’s core affective state; 2) behavioral cues describing facial expressions and bodily gestures, which enable nuanced visual dynamics; 3) contextual modifiers that integrate character‑specific attributes and stylistic choices to ensure output individuality. For instance, to express “nervousness,” the prompt might contain action terms like furrowed brows, shifting gaze, and rubbing hands, while simultaneously incorporating character‑specific language patterns—such as reserved, timid, or outgoing—to produce comprehensive instructional content. The emotion layer does not represent emotional states using continuous dimensional coordinates; instead, it organizes prompt content around discrete emotion categories, with expression refinement achieved through intensity modifiers and behavioral descriptions. The prompt generation process is not solely based on manual composition, but rather integrates ChatGPT’s natural language generation capabilities with pattern-based control strategies. A well-structured prompt design contributes to improving the model’s ability to organize task constraints and enhances the coherence and structural clarity of generated outputs [34]. Initially, a fundamental emotion‑to‑action mapping table is developed by the researchers, which acts as the training basis for prompt templates. Following that, ChatGPT autonomously generates numerous semantically matched behavior descriptions. Through manual checking and repeated optimization, these descriptions are transformed into a transferable prompt repository. This repository facilitates the swift lookup and assembly of prompts tailored to different emotional conditions and user characteristics, leading to marked improvements in both the speed of generation and the degree of personalized output.

Building upon the aforementioned prompt generation mechanism, this study further provides a representative example to illustrate the complete process from user input to the construction of animation generation instructions. The process begins with natural language input and proceeds through sequential stages of emotional semantic parsing, structured information extraction, and prompt construction, ultimately yielding a standardized textual instruction for animation generation. Initially, the user input is subjected to semantic analysis to identify the embedded emotion category, emotional intensity, and expressive tendency. On this basis, user profile information is incorporated to constrain the mode of emotional expression, enabling the extraction of structured semantic units that encompass emotional states, behavioral features, and stylistic preferences. These semantic components correspond to the Emotion Layer, Behavior Layer, and Context Layer within the multi-level encoding framework, thereby providing semantic support for prompt construction.

During the prompt construction stage, semantic information from each layer is combined and reorganized through a hierarchical template to form a text instruction with a complete semantic structure. The degree of elaboration across different semantic layers varies according to the intensity of the input emotion, the complexity of behavioral requirements, and user-specific characteristics. When emotional intensity is higher or more detailed behavioral expression is required, the Behavior Layer is correspondingly refined; when user profiles emphasize stylistic distinctions, the Context Layer is further enriched. These variations are reflected in the dynamic restructuring of the prompt. The complete prompt text and its structural representation for the corresponding example are provided in the Supplementary Material (Example S1 File), along with the associated generation parameter settings to ensure consistency in the generation process.

### 3.2. Personalized modeling mechanism

A personalized modeling mechanism is established in this study, centered around person‑to‑person variations in the expression of emotional animation. Employing ChatGPT to identify and integrate user characteristics, along with Runway ML to produce distinct animation styles, the system supports targeted visual output adjustments through natural language commands and the responses they generate.To enhance the operability of the personalization mechanism and ensure experimental comparability, this study adopts a standardized user profiling approach to structurally summarize variations in personality tendencies, aesthetic preferences, and expressive styles, and examines their influence on animation generation outcomes under consistent emotional conditions.

During the user feature identification stage, a structured description template is designed to construct user profile information across multiple dimensions, including personality traits, aesthetic preferences, perceived motion rhythm, and modes of facial expression. Considering that the primary objective of this study is to evaluate the impact of personalized prompting mechanisms on generation results, rather than to conduct empirical investigations of real user behavior, the user profiles employed in the experiments are constructed based on standardized scenario settings, combined with representative personality characteristics to form typical user archetypes. Examples of such entries include preferences regarding color schemes (warm vs. cool), movement presentation (dramatic or understated), facial expressions (overstated or controlled), and character types (lifelike or animated). ChatGPT performs linguistic condensation and logical arrangement on the gathered text, deriving instructive keyword combinations of significance. This approach circumvents the steep learning curve typical of conventional algorithmic modeling, while boosting expressive precision and generation efficiency via repeated interactive optimization.

In the stage of integrating personality characteristics, the research incorporates the above‑mentioned keywords as prompt terms into the animation generation instructions. These are combined with emotional settings and animation style preferences to form comprehensive language descriptions. When characterizing a composed personality that favors low‑saturation colors and restrained emotional expression, the researchers formulate natural language generation requests that specify slow movement pacing, subtle changes in facial expression, and muted color tones. Such prompts are then revised and refined with the help of ChatGPT to ensure clear structure and adherence to Runway ML’s language recognition requirements.

During the animation generation stage, the research feeds the personalized prompts—already processed by ChatGPT—directly into the Runway ML platform. By interpreting natural language instructions semantically and constructing image sequences, the platform produces animation clips that align with both the intended emotional context and the user’s personality traits. The entire process depends solely on linguistic input to automatically compose and render keyframe scenes, facial expressions, body movements, and color schemes. Beyond emotion recognition, Runway ML also responds to personality‑related information contained within the prompts, applying stylistic regulation across several critical animation dimensions. In terms of facial dynamics, characters exhibit differences in expression intensity, transition frequency, and onset timing depending on the personality setting. For motion rhythm, the generated content adjusts movement speed and keyframe transition rates in accordance with rhythm‑related vocabulary. Regarding visual style, the platform automatically invokes built‑in style templates based on color preferences and character configurations, thereby controlling color composition, brightness contrast, and framing style. Moreover, Runway ML’s generation mechanism synchronizes style control factors between character performances and background design, ensuring motion consistency and temporal smoothness. As a result, animation content generated under identical emotional conditions but with different user profiles demonstrates clearly distinguishable stylistic variations and expressive characteristics, evidencing a robust capacity for personalized output. Considering the inherent stochasticity in text-driven video generation, this study adopts a strategy that combines repeated generation under consistent parameter settings with consistency-based selection, in order to enhance the stability of comparisons across different user profiles. The effectiveness of the personalization mechanism is therefore not dependent on single-instance outputs, but is instead established on the premise that generation results associated with the same user profile exhibit relatively stable stylistic tendencies.

### 3.3. Construction of multi-level encoding strategy

Within the emotional animation generation system developed in this study, the multi‑level encoding strategy functions as a key intermediary mechanism that bridges linguistic prompts and animation generation logic. This strategy adopts a structured, hierarchical approach to parse input sentences, performing layered extraction and nested embedding of emotional semantics, action intentions, and style modifiers to achieve refined, emotion‑driven control.

**Fig 2:**
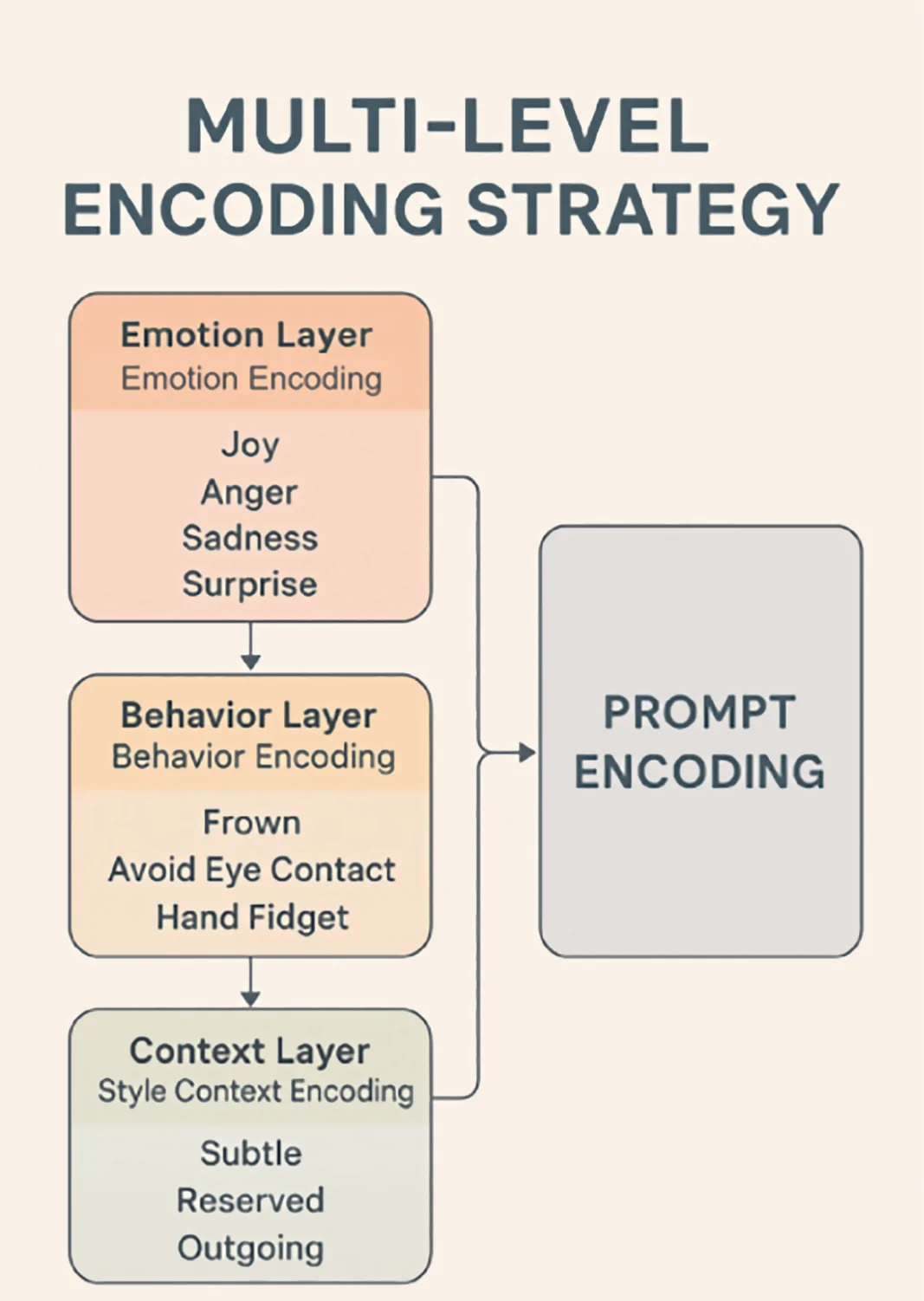
Hierarchical encoding framework for emotion-driven prompt construction.


The encoding framework is structured into three levels: the Emotion Layer, the Behavior Layer, and the Context Layer (Fig 2). At the Emotion Layer, the main emotional state is drawn from the prompts, defining the fundamental goal of the generation process. The Behavior Layer extracts pivotal action words that guide character motions and links them to the platform’s motion‑triggering instructions. The Context Layer records descriptive phrases reflecting expressive styles, cultural contexts, or character attributes, which act as modifiable parameters for controlling stylistic output. During the encoding process, prompts are parsed into structured semantic vector sequences. For the semantic information at the i-th layer, the encoding result is represented as a set of feature vectors:

$$\begin{array}{c} C^{\left(i\right)}=\{c_{\text{1}}^{\left(i\right)},c_{\text{2}}^{\left(i\right)},\ldots ,c_{n}^{\left(i\right)}\}\text{\textbackslash{}hspace\{1em}where\}\;i=1,2,3 \end{array}$$ (1)

Where, *C*^(1)^ denotes the semantic embedding set of the Emotion Layer, *C*^(2)^ represents the semantic embedding set of the Behavior Layer, and *C*^(3)^ corresponds to the semantic embedding set of the Context Layer. The term **c**_k_^(i)^∈$R^{d_{i}}$denotes the feature vector associated with the k-th semantic unit in the i-th layer, where *d_i_* represents the dimensionality of the semantic feature space for that layer. These vectors are obtained by mapping prompts through a semantic encoding model and are used to characterize the structural features of semantic information across different hierarchical levels.

During the prompt construction process, the three layers of semantic information are not simply concatenated but are organized according to a structured relational scheme. The Emotion Layer typically serves as the core semantic foundation of the prompt, explicitly defining the target emotion category and its intensity. Building upon this, the Behavior Layer supplements detailed facial expressions and action features, enabling the animation to exhibit visually perceivable dynamic characteristics. The Context Layer, in turn, refines the overall style and scene atmosphere, ensuring a more complete and coherent visual presentation. Through this hierarchical organization, the prompt establishes clear structural relationships across different semantic dimensions.

As the input emotional state, behavioral complexity, and user profile characteristics vary, the prompt structure undergoes corresponding adjustments. These changes are reflected in the selection scope, descriptive granularity, and combinational patterns of semantic units across different layers, thereby shifting the emphasis of the prompt. When emotional intensity is higher, descriptions associated with the Emotion Layer become more concentrated; when more detailed motion representation is required, the Behavior Layer is further refined with increased descriptive density; and when personalized stylistic requirements are emphasized, the relative contribution of the Context Layer is correspondingly enhanced. These adjustments are manifested during the prompt construction stage, resulting in differentiated distributions of semantic information across layers within the final expression.

On animation generation platforms like Runway ML, the encoded outputs are converted into control commands and fed into the driving module. The three‑layer encoding influences expression parameters, motion paths, and situational performance components, enabling a highly consistent mapping from semantic input to visual output. To handle scenarios involving mixed emotions or varied character styles, the study additionally introduces a dynamic weight adjustment mechanism. This mechanism allows the encoding vectors to expand according to contextual semantic density and dynamically adjusts generation biases, ensuring that the resulting animation remains coherent while exhibiting stylistic differentiation.

### 3.4. Evaluation framework

To enhance the verifiability and standardization of emotion animation generation results, this study constructs an evaluation framework that integrates human ratings with objective quantitative metrics, accompanied by a standardized annotation procedure to ensure consistency and reproducibility of the evaluation outcomes. The framework centers on emotional expression quality and dynamic animation performance, providing a comprehensive assessment of model outputs from both subjective and objective perspectives. In existing research on generative visual content evaluation, Fréchet Inception Distance (FID) is commonly used as a proxy metric for image realism, while Fréchet Video Distance (FVD) is widely adopted to measure distributional similarity in terms of visual quality and temporal consistency in generated videos [35,36]. However, given that the primary focus of this study lies not in general image fidelity or video generation quality, but rather in the accuracy of emotional semantic expression, the coherence of motion logic, and the adaptability of personalized styles, the proposed evaluation framework prioritizes human ratings and task-oriented objective feature indicators.

At the subjective evaluation level, a structured coding scheme is constructed to quantitatively describe animation performance. For the i-th animation sample, its rating vector is defined as:


$$\begin{array}{c} {𝐒}_{i}=\left[s_{i1},s_{i2},s_{i3},s_{i4}\right] \end{array}$$ (2)

where *s*_i1_ denotes emotional expression accuracy, *s*_i2_ represents fine-grained facial expressiveness, *s*_i3_ corresponds to motion logical coherence, and *s*_i4_ indicates temporal consistency. Each dimension is evaluated using a five-point rating scale, defined as follows:


$$\begin{array}{c} s_{ij}\in \left\{1,2,3,4,5\right\} \end{array}$$ (3)

In the specific assessment process, emotional expression accuracy measures the degree of alignment between the generated animation and the target emotion category; fine-grained facial expressiveness evaluates whether dynamic changes in key regions—such as the eyebrows, eyes, and mouth—are clearly represented and exhibit sufficient detail and layering; motion logical coherence assesses whether bodily actions conform to the progression of emotional states and the predefined character settings; and temporal consistency reflects the continuity and stability of transitions between animation frames. By explicitly defining the evaluation criteria for each dimension, different evaluators are able to conduct assessments under a unified standard, thereby improving the reproducibility of the evaluation outcomes.

For the evaluation of personalized expression, to quantify the consistency between generated results and the target user profile, this study introduces a user-profile matching metric, which is defined as:


$$\begin{array}{c} P_{i}=\frac{\text{1}}{M}\sum_{j=1}^{M}p_{ij} \end{array}$$ (4)

where *P*_*i*_ enotes the user-profile matching score for the i-th animation sample, *p*_ij_ epresents the rating assigned by the j-th evaluator for that sample on the dimension of “user-profile consistency,” and M indicates the total number of evaluators.

At the objective quantification level, this study introduces fundamental visual and motion feature metrics to enhance the measurability of the results. Specifically, expression intensity is characterized by the difference between facial feature vectors and a neutral state, which is formulated as:


$$\begin{array}{c} I_{expr}=\frac{\text{1}}{T}\sum_{t=1}^{T}\|{𝐟}_{t}-{𝐟}_{neutral}\| \end{array}$$ (5)

where **f**t denotes the facial feature vector at frame t. Furthermore, posture variation is described using geometric angles formed by keypoints, which is expressed as:


$$\begin{array}{c} \theta =arctan\left(\frac{y_{\text{2}}-y_{\text{1}}}{x_{\text{2}}-x_{\text{1}}}\right) \end{array}$$ (6)

This metric is used to capture variations in motion amplitude under different emotional states or personalized settings. Furthermore, color characteristics are described using pixel-level statistical measures, with variance defined as:


$$\begin{array}{c} \sigma_{c}^{\text{2}}=\frac{\text{1}}{N}\sum_{i=1}^{N}(c_{i}-\overset{―}{c}{)}^{\text{2}} \end{array}$$ (7)

where *c*_i_ denotes the pixel color value and $\overset{―}{c}$ represents the mean value. In addition, to characterize the continuity and stability of animations along the temporal dimension, this study introduces a motion smoothness metric, which is defined as:


$$\begin{array}{c} S_{motion}=\frac{\text{1}}{T-2}\sum_{t=2}^{T-1}\|{𝐱}_{t+1}-2{𝐱}_{t}+{𝐱}_{t-1}\| \end{array}$$ (8)

where **x**_t_ denotes the keypoint vector at frame t. This metric is based on the second-order difference of keypoint motion and is used to quantify the smoothness of action variations; a smaller value indicates more continuous transitions over time, thereby effectively reflecting motion smoothness characteristics. The aforementioned metrics provide quantitative descriptions of animation performance across three dimensions—facial expression, motion dynamics, and visual style—thereby complementing the limitations of purely subjective visual assessment.

In terms of the annotation procedure, this study adopts a multi-evaluator independent rating mechanism. A total of 12 evaluators participated in the experiment, all of whom possess academic backgrounds in animation design or digital media. Prior to the evaluation, all participants were provided with standardized instructions and example-based training to ensure a clear understanding of the criteria for each evaluation dimension. Subsequently, the animation samples were randomly ordered, and all generation condition information was removed to establish a blind evaluation setting. Each evaluator completed the scoring process independently, thereby minimizing potential bias caused by mutual influence. Each animation sample was rated repeatedly by multiple evaluators to ensure the stability and reliability of the results.

To assess the consistency of the ratings, the intra-class correlation coefficient (ICC) is employed, which is defined as:


$$\begin{array}{c} ICC=\frac{MS_{B}-MS_{W}}{MS_{B}+(k-1)MS_{W}} \end{array}$$ (9)

where *MS*_B_ and *MS*_W_ denote the between-group and within-group mean squares, respectively, and k represents the number of evaluators. This metric is used to quantify the degree of agreement among different evaluators, thereby validating the reliability of the subjective assessment.

At the statistical analysis level, the mean and standard deviation of the rating results are computed, and an independent-samples t-test is conducted to examine the significance of differences between different generation strategies, which is expressed as:


$$\begin{array}{c} t=\frac{\mu_{\text{1}}-\mu_{\text{2}}}{\sqrt{\frac{\sigma_{\text{1}}^{\text{2}}}{n_{\text{1}}}+\frac{\sigma_{\text{2}}^{\text{2}}}{n_{\text{2}}}}} \end{array}$$ (10)

Through the construction of the above evaluation framework, this study establishes a unified analytical paradigm that integrates subjective assessment with objective quantification. This enables the performance of emotion-driven animation generation to be systematically validated across multiple dimensions, including emotional expression, motion dynamics, and visual characteristics, thereby effectively enhancing the reliability and interpretability of the research findings.

### 3.5. Experimental setup

**Fig 3:**
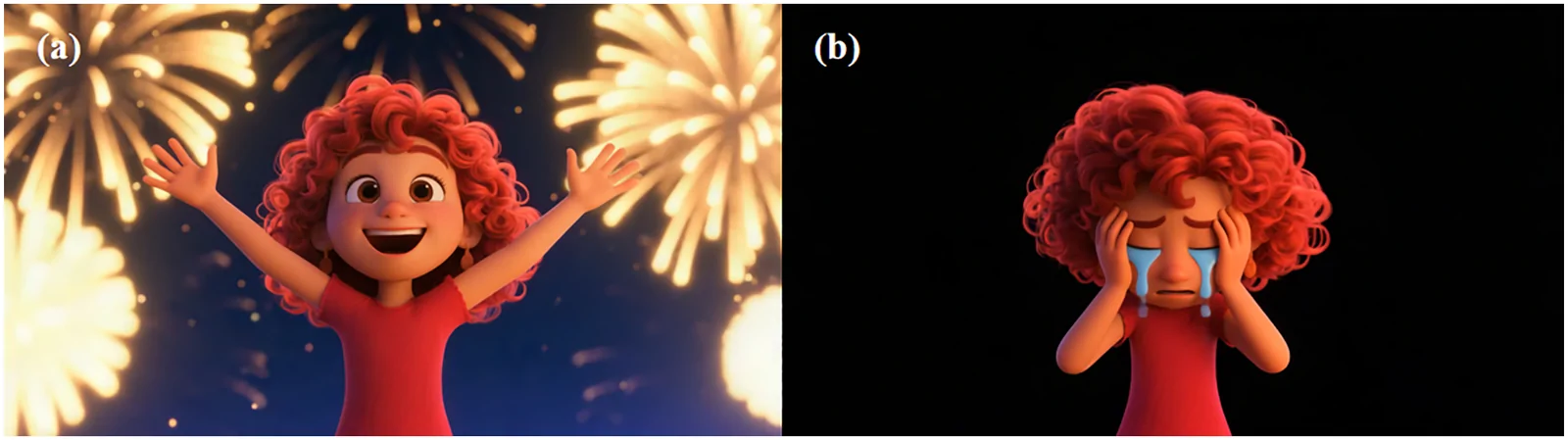
Representative samples of generated animations under different basic emotional conditions. This figure was generated using Runway ML Gen-2 based on author-provided text prompts, with prompt construction assisted by ChatGPT GPT-4o. (a) Prompt for the happiness condition: Emotion: high-intensity happiness; Behavior: broad smile, raised eyebrows, energetic arm movements; Context: bright environment with warm lighting and vivid colors. (b) Prompt for the sadness condition: Emotion: high-intensity sadness; Behavior: teary eyes, lowered eyebrows, noticeable body motion; Context: dim environment with cool tones and low brightness. Both sets of results are generated under identical parameter settings: Runway ML Gen-2 (2025 stable version), 24 fps, 4 s duration, 1024 × 576 resolution, motion strength = 0.6, guidance scale = 7.5, with the negative prompt blurry motion, distorted limbs, unstable background. Each prompt is independently generated five times, and each subfigure presents one representative sample. The displayed results are not randomly selected, but rather the highest-scoring samples chosen from the five independent generations under each condition based on predefined evaluation criteria.

**Fig 4:**
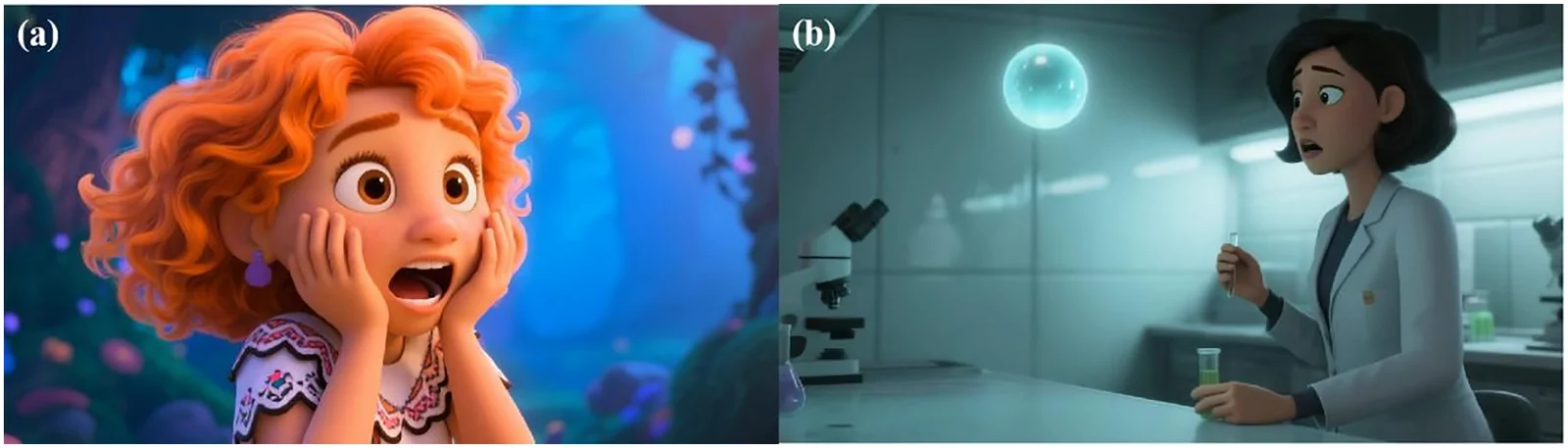
Comparative illustration of differentiated expression under the “surprise” emotion across user profiles. This figure was generated using Runway ML Gen-2 based on author-provided text prompts, with prompt construction assisted by ChatGPT GPT-4o. (a) Prompt for the lively profile: Emotion: high-intensity surprise; Behavior: wide eyes, open mouth, sudden movement; Context: bright dynamic environment with strong contrast. (b) Prompt for the reserved profile: Emotion: high-intensity surprise; Behavior: widened eyes, slight mouth opening, limited motion; Context: softly lit environment with balanced tones. Both sets of results are generated under identical parameter settings: Runway ML Gen-2 (2025 stable version), 24 fps, 4 s duration, 1024 × 576 resolution, motion strength = 0.6, guidance scale = 7.5, with the negative prompt blurry motion, distorted limbs, unstable background. Each prompt is independently generated five times, and each subfigure presents one representative sample. The displayed results are high-scoring samples selected under a unified evaluation framework for each experimental condition, rather than arbitrary selections based on subjective preference.

**Fig 5:**
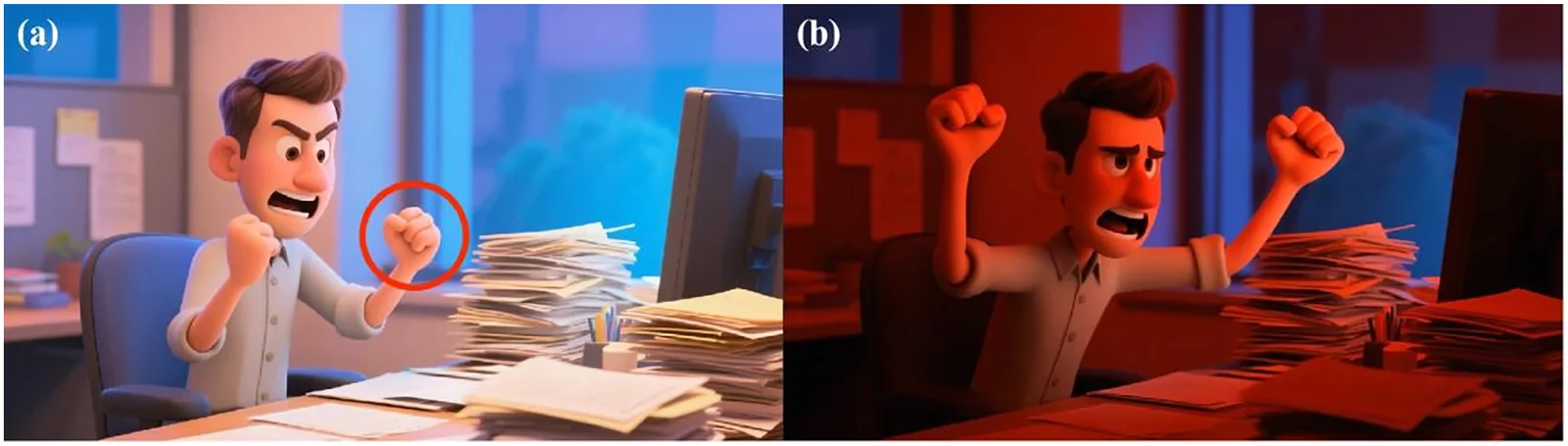
Comparative results of animation generation under different prompt organization strategies for the “anger” emotion. This figure was generated using Runway ML Gen-2 based on author-provided text prompts, with prompt construction assisted by ChatGPT GPT-4o. (a) Without multi-level encoding, using a non-hierarchical prompt: A lively character expressing high-intensity anger with furrowed brows, a clenched jaw, and forceful gestures in a dark environment with high contrast and intense lighting. (b) With multi-level encoding, using a hierarchical prompt: Emotion: high-intensity anger; Behavior: furrowed brows, clenched jaw, forceful gestures; Context: dark environment with high contrast and intense lighting. Both conditions contain identical semantic information, keyword coverage, and descriptive granularity. The only difference lies in the prompt organization format, namely linear versus hierarchical representation. All results are generated under the same parameter settings: Runway ML Gen-2 (2025 stable version), 24 fps, 4 s duration, 1024 × 576 resolution, motion strength = 0.6, guidance scale = 7.5, with the negative prompt blurry motion, distorted limbs, unstable background. Each condition is independently generated five times. Each subfigure presents one representative sample. The displayed results are not randomly selected but are the highest-scoring samples chosen from the five generated outputs under each condition, based on predefined criteria including emotional consistency, motion coherence, and visual integrity.

To enhance reproducibility and transparency, this study standardizes the generation tools, parameter configurations, prompt construction procedures, and result selection criteria. In the text semantic generation stage, ChatGPT is employed to generate descriptions of emotions and behaviors. The specific model version used in the experiments is GPT-4o (OpenAI), accessed on May 15, 2025. ChatGPT GPT-4o was used to assist in emotion analysis, behavioral description generation, and hierarchical prompt refinement based on author-provided task requirements. In the visual generation stage, Runway ML Gen-2 was used as the AI video/image generation tool to produce the experimental animation images shown in Figs 3–5 based on author-provided text prompts. The relevant Terms of Use are available at OpenAI Terms of Use: https://openai.com/policies/row-terms-of-use/ and Runway Terms of Use: https://runwayml.com/terms-of-use. The generated figures were created for research demonstration purposes and are intended to be published under the CC BY 4.0 license in accordance with the journal’s requirements. During the prompt construction process, a fixed task prompt template is consistently applied, without introducing additional system prompts, in order to avoid potential variations caused by different contextual settings. On this basis, the generated content is further constrained using a predefined hierarchical structure, ensuring that the output text maintains a consistent organization across three dimensions: emotional category, behavioral characteristics, and contextual information. This design guarantees the comparability of prompts under different experimental conditions.

In terms of prompt construction, this study adopts a multi-level encoding strategy to divide prompts into three components: the Emotion Layer, the Behavior Layer, and the Context Layer, which are combined through a fixed template. The composition and functions of each layer are summarized in Table 1, while the complete hierarchical prompt template and corresponding rules are provided in Table S1 in S1 File of the Supplementary Material. Specifically, the Emotion Layer defines the emotional category and intensity, the Behavior Layer describes action and facial expression characteristics, and the Context Layer constrains environmental settings and visual style. To establish a comparable baseline, a “non-hierarchical prompt” condition is introduced as a control. Under this condition, the semantic content is identical to that of the hierarchical prompts but is reorganized into a linear sequence. This ensures that both prompt types maintain equivalent coverage of emotional, behavioral, and contextual information, while further controlling for keyword quantity, descriptive granularity, and overall length within a similar range. As a result, the only difference between experimental conditions lies in the prompt organization format—hierarchical structure versus flat linear structure—rather than differences in information richness. Based on this controlled design, the study is able to more accurately isolate and evaluate the effect of the multi-level encoding structure itself on animation generation outcomes, avoiding the confounding influence of longer or more detailed prompts. Concrete examples comparing encoded and non-encoded prompts are provided in Table S2 in S1 File of the Supplementary Material.

**Table 1:** Hierarchical prompt structure for emotion-driven animation generation.

Layer	Component	Function	Example content
Emotion Layer	Emotion type	Defines the target emotional category	happiness
Emotion Layer	Emotion intensity	Specifies the strength of the emotional expression	high intensity
Behavior Layer	Facial expression	Describes facial features associated with the emotion	broad smile, raised eyebrows
Behavior Layer	Body movement	Defines gesture amplitude and motion pattern	energetic arm movements
Context Layer	Environment	Specifies the scene or background setting	bright environment
Context Layer	Visual style	Defines tone, lighting, and atmosphere	warm lighting, vivid colors

### 3.6. Ethics statement

In the process of conducting emotion animation generation based on AIGC tools, beyond technical limitations, potential ethical implications should also be carefully considered. First, generative models may be influenced by the distribution of training data in the mapping between facial expressions and emotional states, which can lead to representational biases across different populations or contexts. Such biases may affect the fairness and consistency of emotional expression. Second, emotional modeling and animation representation may be interpreted differently across cultural contexts. The current prompt construction is primarily grounded in specific linguistic and cultural backgrounds, and its cross-cultural applicability requires further validation. Finally, emotion-driven animation generation technologies may, in practical applications, be used for emotional guidance or even misleading expression. Therefore, it is necessary to establish appropriate usage regulations and constraints tailored to specific application scenarios to ensure responsible deployment.

## 4. Results and analysis

### 4.1. Analysis of expression accuracy

To systematically evaluate the model’s performance in terms of expression accuracy and semantic consistency in basic emotion recognition and animation generation, this study selects two representative fundamental emotional states—happiness and sadness—and constructs standardized process samples from textual prompt input to animated keyframe output. Both sets of samples are generated under identical character settings, background conditions, lighting configurations, and unified generation parameters. The initial frame in all cases features a vibrant fireworks background, with only the emotional input variable adjusted to control for the influence of non-emotional factors on the output. Under each emotional condition, multiple independent generations are conducted using the same prompt, and the results are evaluated and ranked according to a unified evaluation framework. The samples presented in Fig 3 correspond to the highest-scoring and most representative outputs.

As illustrated in Fig 3, under the “happy” condition (Fig 3(a)), the model accurately interpreted the positive emotional semantics embedded in the prompts and, via Runway ML, produced dynamic keyframes that exhibit distinct emotional traits. In the final frame, the character shows a clear smile, with upturned corners of the eyes, elevated eyebrows, and facial muscles in a state of expanded tension. Body movements include high‑energy postures such as leaning the upper torso forward and extending or lifting the arms. The background features a warm‑toned, highly saturated fireworks display, resulting in an overall bright and energetic atmosphere. These outcomes indicate that the system achieves high accuracy and stability in both semantic reconstruction and visual mapping for positive emotions. From the objective quantitative results shown in Table 2, under this emotional condition, expression intensity reaches 0.842, posture variation 0.765, and color variance 1285.34. These values indicate a high level of facial muscle activation, pronounced body movement amplitude, and strong color contrast. This suggests that the system demonstrates a high capability for semantic reconstruction in positive emotion recognition and perceptual mapping.

**Table 2:** Quantitative results of objective features for happiness and sadness emotion animations.

Metric	Happiness	Sadness	t-value	p-value	Cohen’s d
Facial Expression Intensity	0.842	0.413	6.27	<0.001	1.85
Posture Variation	0.765	0.298	5.94	<0.001	1.72
Color Variance	1285.34	642.17	5.21	<0.001	1.51

Under the “sad” scenario (Fig 3(b)), the model again correctly identified the negative emotional cues within the prompt and progressively diminished the energetic expression of the background and motion across the animation sequence. In the concluding frame, the character exhibited constricted facial features—furrowed brows, lowered eyelids, and a downturned mouth. The posture was contracted, and the limb movements were slow or entirely motionless. Meanwhile, the background exhibits a transition from high brightness to lower luminance, ultimately converging toward a darker tone. This visual pattern is consistent with a low-arousal, negative-valence emotional atmosphere. Such background transitions appear to involve a degree of automated completion during the generation process and are not entirely governed by explicit control within the prompts. In contrast, facial expressions, posture variation, and the temporal organization of actions demonstrate more stable and consistent patterns across different emotional conditions. From the quantitative metrics, under this emotional state, expression intensity is 0.413, posture variation 0.298, and color variance 642.17, all of which are significantly lower than those observed in the happiness condition. This reflects more restrained facial expressions, reduced motion amplitude, and diminished color contrast. These results indicate that the model maintains a high level of consistency and precision in both the recognition and visual mapping of sadness-related emotions.

These comparative findings confirm that the proposed hierarchical prompt encoding strategy effectively supports the structured interpretation of emotional meaning, ensuring that the relationships between emotional categories and their visual expressions in animation are preserved. The quantitative results reveal consistent patterns of differences between happiness and sadness across expression intensity, posture variation, and color variance. This indicates that the model not only achieves clear emotional differentiation at the visual level, but also demonstrates strong discriminative capability within the objective feature space. Further statistical analysis shows that differences across all metrics under different emotional conditions are statistically significant (p < 0.001), with large effect sizes. This confirms that distinct emotional states exhibit substantial and practically meaningful differences in terms of facial expression intensity, posture dynamics, and visual characteristics. Once the parsing was complete, Runway ML precisely called upon the appropriate expression templates, motion patterns, and color palettes for each emotion, thereby significantly boosting the overall expressive power of emotion‑driven animations. Further temporal examination of the keyframes revealed a high degree of synchronization between facial expressions and bodily movements, with emotional features preserved smoothly and naturally across the entire animated sequence. This underscores the model’s advantage in both learning and generating consistent expressive dynamics.

### 4.2. Analysis of personalization performance differences

This section conducts a comparative analysis of animation generation differences across user profiles under a unified emotional input of “surprise.” Two representative profiles—“lively” and “reserved”—are selected. Multiple independent generations are performed under consistent prompt semantics and unified generation parameters, and the results are evaluated and screened based on a standardized evaluation framework. Fig 4 presents representative high-scoring samples under each condition. Based on these results, a comparative analysis is carried out from the perspectives of stylistic presentation, facial expression, and motion rhythm, in order to assess the model’s adaptability in emotion animation generation under different user characteristics.

For the “lively” profile (Fig 4(a)), the generated animation displays a distinctly high‑energy and outwardly expressed form of surprise. The character’s eyes are wide open, with dilated pupils fixed straight ahead; the eyebrows are raised prominently toward the forehead; and the mouth forms an exaggerated “O” shape. Both hands rapidly rise to the sides of the face with a pronounced outward stretch, elbows flare widely, shoulders lift slightly, and the upper body leans forward—conveying an immediate, intense emotional reaction. The motion rhythm is quick, and the synchrony between facial and hand movements is high, producing a continuous and coherent surprise dynamic. In terms of color, the overall scene adopts a warm, high‑saturation palette. The bright background incorporates softly blurred, dreamlike elements, further amplifying the emotional tension and impact while enhancing the animation’s lively and exuberant visual appeal. As shown in Table 3, under this profile, expression intensity reaches 0.912, posture variation 0.868, and color variance 1496.27. These results indicate a high level of facial muscle activation, pronounced motion amplitude, and strong color contrast, collectively reflecting a typical high-arousal expressive pattern.

**Table 3:** Quantitative results of visual expression features under different user profiles for the “surprise” emotion.

Metric	Lively Profile	Calm Profile	t-value	p-value	Cohen’s d
Facial Expression Intensity	0.912	0.534	6.13	<0.001	1.78
Posture Variation	0.868	0.412	5.76	<0.001	1.65
Color Variance	1496.27	821.45	5.08	<0.001	1.49

In contrast, the “reserved” profile presents a more subdued and controlled expression of surprise (Fig 4(b)). Although the basic features of wide‑open eyes and raised eyebrows remain, the mouth opens only slightly, with little tension in the lips. The character holds a test tube at chest height, with movement amplitude markedly lower than in the “lively” profile, and maintains a relatively stable upper‑body posture throughout the emotional expression. The body leans slightly backward, while subtle tension in the shoulders and neck visually conveys a sense of caution and deliberation. The background shows a low‑saturation, cool‑toned laboratory environment with a clean, orderly composition, focused light sources, and a cooler color temperature, reinforcing a rational and professional atmosphere that makes the emotional expression more restrained and contextually suitable. From the quantitative metrics, under this profile, expression intensity is 0.534, posture variation is 0.412, and color variance is 821.45. All values are significantly lower than those of the lively profile, reflecting more restrained facial expressions, reduced motion amplitude, and lower visual contrast.

Taken together, the comparison demonstrates that the integration of multi‑level prompt encoding with personalized modeling mechanisms can precisely capture subtle differences between user profiles under identical emotion‑driven conditions. The quantitative results reveal a consistent pattern of differences between the lively and reserved profiles across expression intensity, posture variation, and color variance. This indicates that the personalized modeling mechanism not only enables clear stylistic differentiation at the visual level, but also produces distinct separations within the objective feature space. Further statistical analysis shows that the differences between user profiles across all evaluation metrics are statistically significant (p < 0.001), with large effect sizes. This demonstrates that personalized characteristics have a substantial impact on animation expression in terms of facial representation, motion amplitude, and visual style. Such variation reflects both the model’s precise adjustment of emotional intensity and expressive style, and its adaptable performance across visual aesthetics, motion reasoning, and scene composition. This provides strong support for achieving personalized output in the generation of emotion‑driven animations.

To further validate the discriminative capability across different user profiles, a user profile matching score was introduced in addition to the original visual feature metrics, and statistical analysis was conducted based on multiple independent generation results. The results are presented in Table 4. Overall, consistent patterns of differences are observed across all evaluation metrics for different user profiles. The statistical analysis indicates that the lively and reserved profiles exhibit significant differences in both the user profile matching score and all visual feature metrics (p < 0.001), with large effect sizes. This demonstrates that the model is capable of achieving stable and distinct expressive differentiation under varying personality configurations. Specifically, the lively profile shows higher levels of facial activation, greater motion amplitude, and stronger color contrast, whereas the reserved profile is characterized by more restrained dynamic variation and a more controlled visual style, aligning well with their respective profile definitions. Analysis of multiple independent generation results further shows that the variability of each metric within the same user profile condition remains relatively low, indicating strong stability in repeated generation. In addition to the two representative profiles highlighted in this study, additional user types constructed from diverse combinations of personality traits were also evaluated under the same experimental framework. The observed differentiation patterns remain consistent with those reported in the table, further supporting the model’s capability to distinguish across a wide range of user characteristics.

**Table 4:** Quantitative evaluation of personalization consistency and profile separability under the “surprise” condition.

Metric	Lively Profile	Calm Profile	t-value	p-value	Cohen’s d
Persona Alignment	4.34 ± 0.31	3.21 ± 0.28	6.18	<0.001	1.74
Facial Expression Intensity	0.91 ± 0.04	0.53 ± 0.05	6.07	<0.001	1.71
Posture Variation	0.87 ± 0.05	0.41 ± 0.06	5.69	<0.001	1.60
Color Variance	1496.27 ± 96.42	821.45 ± 88.37	5.14	<0.001	1.45

### 4.3. Effectiveness validation of encoding strategy

This section conducts a comparative analysis of animation performance under two generation modes—without multi-level encoding and with the multi-level encoding strategy—using a unified emotional input of “anger.” To ensure that the comparison reflects the effect of prompt structure itself, both generation modes adopt prompts with identical semantic content, strictly controlling emotional information, behavioral descriptions, and contextual expressions, while differing only in their organizational form, namely linear versus hierarchical representation. Each condition is subjected to multiple independent generations under consistent parameter settings, and the results are evaluated and selected based on the predefined evaluation framework. As illustrated in Fig 5, representative high-scoring samples under each condition are presented to demonstrate the impact of different prompt structures on animation detail and temporal coherence.

In the “no multi‑level encoding” scenario (Fig 5(a)), the resulting animation depicts anger using facial expressions that include heavily lowered brows, enlarged eyes, and an open mouth as if shouting, yet the rendering of details is rather crude. Regarding hand movements, the character’s fist is made by pressing the four fingers together with the thumb wrapped on the outside; the motion range is small, and there is no gradual progression from a slight shake to a tight fist, so the change in anger intensity is insufficiently pronounced. The temporal continuity of bodily actions is poor, with noticeable jump‑like artifacts during transitions of arm and shoulder postures, and a missing dynamic arc from emotional preparation to full outburst. The background stays with standard scene illumination and lacks visual elements that would heighten the emotional tone, resulting in limited immersive quality. As shown in Table 5, under this configuration, the expression intensity reaches 0.822, posture variation 0.792, and color variance 908.45. These results indicate that, although a certain level of emotional expressiveness is achieved, the overall performance tends to reflect momentary amplification rather than a structured representation.

**Table 5:** Quantitative results of visual expression features under different encoding strategies for the “anger” emotion.

Metric	Without Hierarchical Encoding	With Hierarchical Encoding	t-value	p-value	Cohen’s d
Facial Expression Intensity	0.822	0.898	2.42	<0.01	0.98
Posture Variation	0.792	0.836	3.87	<0.01	0.96
Color Variance	908.45	1412.38	3.21	<0.001	1.54

In the “multi‑level encoding strategy” scenario (Fig 5(b)), the prompts underwent hierarchical parsing across the emotional, behavioral, and contextual layers, integrating descriptive elements such as “knitted brows and a fixed glare,” “gritted teeth,” “fists slowly tightening and then abruptly rising,” and “a forward lean with power coming from the shoulders.” The generated output presents a more pronounced dynamic evolution in facial detail—progressing from an initial frown to gradually clenched teeth and lowered mouth corners—in a logically ordered sequence of facial muscle movements. Regarding hand gestures, the character not only curls all five fingers tightly into the palm, with the thumb securely enclosing the other fingers, but also shows nuanced changes in squeezing force, steadily escalating the action to enhance both power and emotional intensity. The pacing of body movements and their synchronization with upper‑body motion are significantly better, featuring a smooth and uninterrupted shift from energy accumulation to explosive release, with inter‑frame transitions appearing seamless and natural. The background lighting adopts a more theatrical style, employing contrasts of deep red and dark gray to heighten the tense, suffocating atmosphere, thereby making the visual portrayal of anger more vivid and compelling. From the quantitative metrics, under this strategy, expression intensity reaches 0.898, posture variation increases to 0.836, and color variance rises to 1412.38. These results indicate that, while effectively controlling expression amplitude, the model enhances motion coordination and visual consistency, enabling emotional expression to transition from abrupt changes to a more gradual progression.

Taken together, while the animations generated without multi‑level encoding are capable of depicting anger in a general sense, they fall short in terms of gradual detail refinement, accuracy of hand actions, and continuity over time. In contrast, the multi‑level encoding approach, via its hierarchical prompt structure, combines variations in emotional intensity, nuanced detailing, and environmental mood, thereby realizing a strong alignment between linguistic meaning and visual results. The statistical analysis results indicate that the hierarchical encoding strategy achieves significant improvements in posture variation, expression intensity, and color-related metrics (p < 0.01), with effect sizes at a medium level or above. This demonstrates that the proposed strategy effectively enhances motion expressiveness, visual style consistency, and emotional representation in generated animations. These findings suggest that the multi-level encoding mechanism improves motion logic and visual coherence while maintaining the accuracy of emotional expression. By enabling hierarchical transmission of emotional information within prompts and leveraging the Runway ML platform to achieve a high-consistency mapping from semantics to visual output, the proposed approach significantly enhances the precision and realism of facial expressions, motion dynamics, and contextual representation, thereby providing critical support for high-quality emotion animation generation.

### 4.4. Comparative analysis of generation performance

To systematically evaluate the performance of the proposed method in emotion animation generation tasks, three representative approaches—linear prompting, optimized prompt engineering, and multimodal prompting—were selected as baselines for comparison. A quantitative analysis was conducted under a unified experimental setup and evaluation framework. As shown in Table 6, significant differences are observed across methods on all evaluation metrics. The linear prompting method demonstrates relatively weak overall performance, with low scores across all dimensions, indicating that reliance on simple semantic descriptions is insufficient for achieving stable and fine-grained emotional expression. The optimized prompt engineering approach improves emotion accuracy and facial detail by enhancing keyword specificity and semantic richness; however, it still exhibits limitations in motion logic and temporal consistency. The multimodal prompting method further integrates emotional, behavioral, and contextual information, resulting in notable improvements across all metrics, particularly in temporal consistency. Nevertheless, its performance still shows certain fluctuations in fine-grained control and dynamic stability.

**Table 6:** Quantitative comparison of different methods.

Method	Emotion Accuracy	Facial Detail	Motion Logic	Temporal Consistency	Motion Smoothness
Linear Prompt	3.08	2.95	2.87	3.02	0.089
Advanced Prompt Engineering	3.76	3.58	3.49	3.62	0.068
Multimodal Prompt	3.98	3.81	3.72	3.86	0.059
Proposed Method	4.36	4.28	4.21	4.33	0.041

In comparison, the proposed method achieves the best performance across all evaluation dimensions. Specifically, emotion accuracy reaches 4.36, facial micro-expression detail 4.28, motion logic 4.21, and temporal consistency 4.33, all of which are significantly higher than those of the baseline methods. These results indicate that the multi-level encoding strategy effectively enhances the precision of emotional semantic parsing and the consistency of visual expression. From the perspective of the encoding mechanism, this performance improvement primarily stems from the structured modeling and coordinated fusion of multi-layer semantic information. In terms of temporal consistency, the proposed method separates the encoding of emotion and behavior layers and integrates them within a unified feature space, allowing emotional state transitions to be continuously propagated along the temporal axis. This design effectively mitigates the frame-to-frame fluctuations caused by semantic entanglement in conventional prompting methods. In contrast, linear prompting and multimodal prompting approaches typically rely on sequential text-driven generation without explicit hierarchical constraints, making emotional expression prone to instability or abrupt transitions over time, which results in lower temporal consistency scores. Regarding motion logic, the multi-level encoding strategy models behavioral semantics as an independent layer, enabling motion generation to be guided by explicit behavioral descriptions rather than implicit inference from global semantics. This significantly improves the alignment between motion patterns and emotional states. Experimental results show that motion logic improves from 3.72 to 4.21, demonstrating that structured behavioral encoding effectively reduces unreasonable motion combinations and semantic mismatches. By contrast, existing methods, lacking explicit behavioral layer modeling, often produce inconsistencies between motion expression and emotional intent. In terms of facial micro-expression detail, the independent encoding of the emotion layer allows key facial features to be accurately parsed and consistently mapped to the generative model, thereby enhancing both the granularity and discriminability of facial expressions. Traditional prompting methods, however, tend to embed emotional information within holistic textual descriptions, lacking fine-grained control over local features and resulting in comparatively coarse facial detail. Consequently, the proposed method achieves a score of 4.28 on this metric, significantly outperforming the baseline approaches.

In terms of objective metrics, motion smoothness decreases from 0.089 in the baseline method to 0.041, indicating a significant improvement in temporal continuity and stability of the generated animations. Overall, as the prompt structure evolves from single-layer descriptions to multi-layer semantic encoding, the quality of animation generation shows a consistent upward trend. The proposed method, through the structured modeling of emotion, behavior, and contextual information, achieves a higher degree of semantic consistency and dynamic coordination, thereby outperforming existing approaches in overall generation performance.

### 4.5. Inter-rater reliability analysis

To validate the reliability and consistency of subjective evaluation results, this study conducted an Intraclass Correlation Coefficient (ICC) analysis on the ratings provided by multiple evaluators. As shown in Table 7, all evaluation dimensions achieved relatively high ICC values. Specifically, emotion expression accuracy reached 0.89, temporal consistency 0.87, facial micro-expression detail 0.86, and motion logic 0.85. Overall, all ICC values exceeded 0.80, indicating a high level of agreement among evaluators across all dimensions and demonstrating that the evaluation process is both stable and reliable.

**Table 7:** Inter-rater reliability results.

Evaluation Dimension	ICC
Emotion Accuracy	0.89
Facial Detail	0.86
Motion Logic	0.85
Temporal Consistency	0.87

Further analysis reveals that emotion expression accuracy and temporal consistency exhibit comparatively higher ICC values, suggesting that evaluators share more consistent criteria when assessing emotional category matching and temporal continuity in animations. This also indirectly reflects the strong stability of the generated results in terms of overall emotional expression and temporal performance. In contrast, facial micro-expression detail and motion logic show slightly lower ICC values, although they still remain within the high-consistency range. This indicates a certain degree of subjectivity in evaluating these dimensions, primarily due to differences in evaluators’ sensitivity to subtle facial variations and the plausibility of motion patterns. Nevertheless, their consistency levels remain sufficient to support the validity of the evaluation results. The ICC analysis thus confirms that the evaluation framework proposed in this study demonstrates strong reliability and reproducibility under multi-rater conditions.

## 5. Discussion

The study introduces an innovative framework for emotion‑driven animation generation that combines ChatGPT and Runway ML, establishing a complete closed‑loop process of “emotion identification, prompt construction, and animation synthesis.” By integrating personalized modeling techniques and a hierarchical encoding strategy, the framework enhances both the consistency of emotional expression and the adaptability to individual users within animated outputs. Experimental results show that the proposed method successfully captures emotional meaning from user inputs and produces stylized visual content tailored to different user characteristics and prompt designs, exhibiting robust performance in terms of precision of expression and personalization control. The multi-level encoding–driven mechanism constructed in this study is not only applicable to emotion animation generation tasks, but also demonstrates potential value across broader animation production workflows. Specifically, the proposed approach provides structured semantic support for key stages such as script generation, character behavior design, and emotion-driven interactive animation, enabling a transition from traditional experience-driven creation toward semantically guided generation. Furthermore, in application scenarios including virtual companionship, digital human interaction, and immersive storytelling, this method enhances the controllability of emotional expression and the adaptability to individual user profiles, thereby improving the consistency of user experience and the overall sense of immersion.

From the perspective of affective computing, the multi-level encoding structure proposed in this study corresponds, to a certain extent, to the mapping process from internal emotional states to externalized expressions. The emotion layer captures the categorical nature of emotions and their intensity, the behavior layer reflects the outward manifestation of emotions through actions and facial expressions, and the context layer further modulates stylistic characteristics and situational appropriateness. This hierarchical structure aligns with the fundamental principles of multidimensional emotion representation in affective computing, where emotions are not treated as single labels but are expressed through the coordinated interaction of multiple channels. In this regard, the present study provides an operational pathway for establishing a structured mapping from emotional semantics to visual expression.

Based on the results analysis, this study further summarizes several cases where the generated outputs are less satisfactory. In a minority of instances, discrepancies are observed between emotional expression and motion performance. Under conditions of high emotional intensity or complex personalized settings, certain samples exhibit insufficient facial variation or mismatched motion rhythms. These issues primarily stem from potential local semantic conflicts arising during the multi-layer semantic composition of prompts, which may interfere with the model’s ability to accurately parse key information. In addition, the multi-level encoding strategy shows a degree of instability across different semantic layers. When emotional and behavioral information are simultaneously emphasized, it may introduce interference that affects the overall consistency of expression. Moreover, AIGC-based generation models inherently exhibit stochasticity under the current experimental settings, which also contributes to variability in the generated results. Therefore, the proposed method still presents certain limitations in scenarios involving complex emotional expression and high degrees of personalized control, with a strong dependence on the structural rigor and semantic consistency of prompts. Future work could focus on optimizing cross-layer semantic coordination mechanisms and introducing more stable generation control strategies to enhance the model’s robustness and generalization capability in complex scenarios.

Our approach demonstrates distinct innovations in both methodology and mechanism design relative to existing research. Le et al. [37] proposed a Transformer‑based animation generation framework that converts emotion labels into image sequences, but their system relies on predefined language corpora and fixed style templates, which restricts its adaptability to individual user variations. In contrast, the current study leverages ChatGPT for dynamic prompt generation and contextual analysis, significantly boosting semantic flexibility and allowing precise expression in multi‑character and multi‑style contexts. Zhao and Zhang [38] presented a multimodal emotion‑driven animation framework for virtual characters that emphasizes enhanced realism via facial motion capture and speech synchronization. Even though their method excels at reconstructing dynamic facial expressions, its data‑driven nature still depends on high‑quality annotated samples and lacks thorough integration with text‑based generative tools. Comparatively, our end‑to‑end AIGC pipeline enables animation generation purely from natural language input, bypassing the need for sensing hardware or large training datasets, thus improving ease of use and generalizability. Ji et al. [39] investigated style transfer mechanisms in emotion‑driven animation by manipulating style embedding vectors to govern visual outputs. Although their technique shows strong stylistic expressiveness, it does not incorporate fine‑grained modeling at the emotional hierarchy level. By contrast, our multi‑level encoding strategy structurally breaks prompts down into emotion, behavior, and context layers, and dynamically adjusts embedding weights to control emotional intensity. This approach not only improves temporal coherence and scene atmosphere but also builds a high‑fidelity mapping mechanism between emotional input and visual output.

To clearly distinguish this study from existing application-oriented research based on tool invocation, its primary contributions can be summarized as follows. First, a three-stage closed-loop framework for emotion-driven animation generation is constructed, enabling a systematic mapping from emotional semantics to visual expression. Second, a hierarchical prompt construction method is proposed to structurally organize emotion, behavior, and contextual information, thereby improving the completeness and controllability of semantic representation. Third, a multi-level encoding strategy is designed to achieve coordinated regulation across different semantic layers, enhancing the consistency of generated results in terms of facial expressions, motion dynamics, and temporal coherence. Fourth, a personalized modeling mechanism is introduced, allowing user-specific characteristics to participate in the generation process and enabling differentiated expression.

Despite the promising performance of the proposed method in terms of emotional consistency and personalized generation, several limitations remain. First, the framework relies on external generative platforms such as Runway ML and ChatGPT, whose underlying model architectures and generation strategies may evolve with version updates, potentially affecting the stability of outputs. Under the current experimental conditions, identical prompts may still yield variations in generated animations at different time points, which limits full reproducibility. Second, as the primary control interface, prompts are inherently constrained by the expressive capacity of natural language, making it difficult to impose fine-grained control over the underlying generative mechanisms. Therefore, the results should be interpreted as validation within a specific platform-dependent context. Finally, conclusions regarding personalized expression are primarily derived from structured analysis and quantitative evaluation metrics, lacking support from large-scale subjective user studies. As such, the external validity at the level of user perception remains to be further verified.

## 6. Conclusion

This study developed a prompt‑controlled generation method that improves the emotional fidelity and stylistic consistency of animated content through semantic hierarchical encoding and user profile integration. By leveraging the collaborative interaction between a language generation tool and a dynamic visual platform, the proposed method enables an automated “text‑to‑emotion‑to‑animation” pipeline that requires no programming. Experimental results show that, regarding expression accuracy, the model’s outputs for the two basic emotions “happiness” and “sadness” achieved a high degree of fidelity to the emotional semantics conveyed by the prompts across multiple dimensions, including facial expressions, motion performance, and color style. Notably, in the “sadness” animation, a highly saturated fireworks background gradually faded to a completely black background over time, while facial and bodily movements remained highly synchronized, fully demonstrating the model’s semantic decoding ability and its consistency between image and expression.For personalized animation generation, under the “surprise” emotion, the animation for the “active” user profile showed both hands rapidly raised near the cheeks with a highly saturated, bright background, whereas the animation for the “calm” user profile showed both hands holding a test tube at chest height, featuring a cooler, low‑saturation color scheme, a more restrained overall posture, and a background conveying a rational atmosphere. Comparative results indicate that the model can accurately recognize and map differences in movement amplitude, color style, and rhythm perception between user profiles, achieving a precise match between animation style and character setting.In the validation of the multi‑level encoding strategy, the comparative experiment under the “anger” emotion demonstrated that, after adopting a layered encoding structure, the generated animation was significantly superior to the non‑encoded condition in terms of facial detail, action logic, and temporal coherence. Under the encoding condition, expressions progressed from frowning to clenching teeth; arms and shoulders exerted force in coordination; and hand gestures changed from a fist formed by pressing four fingers together with the thumb wrapped outside to one with all five fingers curled tightly into the palm, resulting in a more compact and forceful action. These improvements markedly enhanced the power and realism of the anger expression, highlighting the key role of the multi‑level prompt structure in driving richness and naturalness in emotional animation.

The emotion‑driven animation generation method introduced in this work exhibits strong semantic accuracy in its outputs, adaptive responsiveness to individual user characteristics, and robust structural control enabled by hierarchical encoding. Future research can be further extended and deepened along several directions [40]. First, it is necessary to explore control mechanisms beyond prompt-driven paradigms. By introducing controllable generative models or parameterized regulation strategies, emotional semantics can be directly associated with key variables in the generation process, thereby reducing reliance on natural language descriptions and improving the stability and reproducibility of generated results. Second, an explicit mapping between emotional semantics and visual expression can be further constructed. For instance, by establishing structured control parameters or intermediate representation layers, fine-grained regulation of facial expression intensity, motion rhythm, and visual style can be achieved, enhancing both the interpretability and controllability of the generation process. Third, future studies may incorporate multimodal input signals, such as vocal features or physiological data, to enable more accurate recognition and dynamic modeling of emotional states, thereby improving the realism and adaptability of emotional expression. At the evaluation level, it is necessary to conduct large-scale subjective experiments involving real users. By integrating user perception data, the effectiveness of personalized generation can be systematically validated, further strengthening the external validity and practical applicability of the research findings.

## Supporting information

S1 File Supplementary Material Section S1 and S2, Table S1-S5. (DOCX)
