# Supplementary material for: Research on emotion animation generation based on AIGC tools: Personalized expression and multi-level encoding strategy
Source: PLoS One. 2026 Aug 25;21(8):e0356895. doi: 10.1371/journal.pone.0356895 (PMC13505892; doi:10.1371/journal.pone.0356895)
Supplement: Supplementary file 1 [file pone.0356895.s001.docx]

**Supplementary Material**

*Prompt Library, Mapping Table, and Generation Metadata*

This Supplementary Material provides the complete prompt templates, emotion–action mapping, generation settings, and full prompt set used in the study to ensure reproducibility. All prompts follow a standardized hierarchical structure (Emotion–Behavior–Context), and each prompt was executed multiple times under identical settings.

# Example S1. Prompt Design and Coverage

A total of 16 prompts were constructed, covering 4 emotion categories (happiness, sadness, anger, surprise), 2 intensity levels (medium, high), and 2 personality profiles (lively, reserved).

## Table S1. Hierarchical prompt structure

| Layer | Component | Function | Example |
| --- | --- | --- | --- |
| Emotion Layer | Emotion type | Defines emotional category | happiness |
| Emotion Layer | Intensity | Defines strength level | high |
| Behavior Layer | Facial expression | Defines facial features | broad smile |
| Behavior Layer | Body movement | Defines motion pattern | energetic arms |
| Context Layer | Environment | Defines scene setting | bright background |
| Context Layer | Visual style | Defines tone and lighting | warm lighting |

## Table S2. Hierarchical vs Non-hierarchical prompts

| Type | Prompt |
| --- | --- |
| Hierarchical Prompt | Emotion: high-intensity happiness; Behavior: broad smile, raised eyebrows, energetic arm movements; Context: bright environment with warm lighting and vivid colors. |
| Non-hierarchical Prompt | A character expressing high-intensity happiness with a broad smile, raised eyebrows, and energetic arm movements, in a bright environment with warm lighting and vivid colors. |

The hierarchical and non-hierarchical prompts contain identical semantic content, including emotion, behavior, and context descriptions, with comparable length and level of detail. The only difference lies in their structural organization (layered vs. linear format).

## Table S3. Generation settings

| Parameter | Value |
| --- | --- |
| Text model | GPT-4o (OpenAI), accessed May 15, 2025 |
| Video model | Runway ML Gen-2 (2025 stable version) |
| FPS | 24 |
| Duration | 4 s |
| Resolution | 1024×576 |
| Motion strength | 0.6 |
| Guidance scale | 7.5 |
| Negative prompt | blurry motion, distorted limbs, unstable background |
| Generations per prompt | 5 |

# Table S4. Complete paired prompt set with controlled semantic equivalence (16 conditions)

| Emotion | Intensity | Profile | Hierarchical Prompt | Non-hierarchical Prompt |
| --- | --- | --- | --- | --- |
| Happiness | High | Lively | Emotion: high-intensity happiness; Behavior: broad smile, raised eyebrows, energetic arm movements; Context: bright environment with warm lighting and vivid colors. | A lively character expressing high-intensity happiness with a broad smile, raised eyebrows, and energetic arm movements in a bright environment with warm lighting and vivid colors. |
| Happiness | High | Reserved | Emotion: high-intensity happiness; Behavior: gentle smile, slight eyebrow lift, minimal movement; Context: softly lit warm environment with muted colors. | A reserved character expressing high-intensity happiness with a gentle smile, slight eyebrow lift, and minimal movement in a softly lit warm environment with muted colors. |
| Happiness | Medium | Lively | Emotion: moderate happiness; Behavior: relaxed smile, light arm movement, open posture; Context: bright environment with balanced lighting and soft colors. | A lively character expressing moderate happiness with a relaxed smile, light arm movement, and an open posture in a bright environment with balanced lighting and soft colors. |
| Happiness | Medium | Reserved | Emotion: moderate happiness; Behavior: subtle smile, slight facial movement, calm posture; Context: soft-light environment with neutral tones. | A reserved character expressing moderate happiness with a subtle smile, slight facial movement, and a calm posture in a soft-light environment with neutral tones. |
| Sadness | High | Lively | Emotion: high-intensity sadness; Behavior: teary eyes, lowered eyebrows, noticeable body motion; Context: dim environment with cool tones and low brightness. | A lively character expressing high-intensity sadness with teary eyes, lowered eyebrows, and noticeable body motion in a dim environment with cool tones and low brightness. |
| Sadness | High | Reserved | Emotion: high-intensity sadness; Behavior: drooping eyelids, slight frown, minimal movement; Context: dark environment with cool tones and low saturation. | A reserved character expressing high-intensity sadness with drooping eyelids, a slight frown, and minimal movement in a dark environment with cool tones and low saturation. |
| Sadness | Medium | Lively | Emotion: moderate sadness; Behavior: slow movement, mild frown, lowered posture; Context: dim environment with soft cool lighting. | A lively character expressing moderate sadness with slow movement, a mild frown, and a lowered posture in a dim environment with soft cool lighting. |
| Sadness | Medium | Reserved | Emotion: moderate sadness; Behavior: subtle expression, minimal facial change, still posture; Context: low-light environment with muted tones. | A reserved character expressing moderate sadness with subtle expression, minimal facial change, and a still posture in a low-light environment with muted tones. |
| Anger | High | Lively | Emotion: high-intensity anger; Behavior: furrowed brows, clenched jaw, forceful gestures; Context: dark environment with high contrast and intense lighting. | A lively character expressing high-intensity anger with furrowed brows, a clenched jaw, and forceful gestures in a dark environment with high contrast and intense lighting. |
| Anger | High | Reserved | Emotion: high-intensity anger; Behavior: tight facial muscles, narrowed eyes, restrained movement; Context: dim environment with shadowed lighting and cool tones. | A reserved character expressing high-intensity anger with tight facial muscles, narrowed eyes, and restrained movement in a dim environment with shadowed lighting and cool tones. |
| Anger | Medium | Lively | Emotion: moderate anger; Behavior: visible irritation, tense expression, active gestures; Context: slightly dark environment with moderate contrast. | A lively character expressing moderate anger with visible irritation, a tense expression, and active gestures in a slightly dark environment with moderate contrast. |
| Anger | Medium | Reserved | Emotion: moderate anger; Behavior: controlled tension, slight frown, minimal movement; Context: low-light environment with soft shadowing. | A reserved character expressing moderate anger with controlled tension, a slight frown, and minimal movement in a low-light environment with soft shadowing. |
| Surprise | High | Lively | Emotion: high-intensity surprise; Behavior: wide eyes, open mouth, sudden movement; Context: bright dynamic environment with strong contrast. | A lively character expressing high-intensity surprise with wide eyes, an open mouth, and sudden movement in a bright dynamic environment with strong contrast. |
| Surprise | High | Reserved | Emotion: high-intensity surprise; Behavior: widened eyes, slight mouth opening, limited motion; Context: softly lit environment with balanced tones. | A reserved character expressing high-intensity surprise with widened eyes, a slightly open mouth, and limited motion in a softly lit environment with balanced tones. |
| Surprise | Medium | Lively | Emotion: moderate surprise; Behavior: quick reaction, raised eyebrows, light movement; Context: bright environment with moderate contrast. | A lively character expressing moderate surprise with a quick reaction, raised eyebrows, and light movement in a bright environment with moderate contrast. |
| Surprise | Medium | Reserved | Emotion: moderate surprise; Behavior: subtle expression, slight eyebrow raise, minimal movement; Context: neutral environment with soft lighting. | A reserved character expressing moderate surprise with subtle expression, a slight eyebrow raise, and minimal movement in a neutral environment with soft lighting. |

# Table S5. Emotion–action mapping used for prompt construction

| Emotion | Intensity | Facial Expression | Body Action | Visual Cue |
| --- | --- | --- | --- | --- |
| Happiness | High | broad smile, raised eyebrows | energetic arm movements | bright colors, warm lighting |
| Happiness | Medium | gentle smile | relaxed gestures | soft lighting |
| Sadness | High | teary eyes, drooping eyelids | slow movement, lowered posture | dim background, cool tones |
| Sadness | Medium | slight frown | minimal motion | muted colors |
| Anger | High | furrowed brows, tense muscles | forceful gestures | dark tones, high contrast |
| Anger | Medium | tight facial expression | controlled movement | shadowed lighting |
| Surprise | High | wide eyes, open mouth | sudden movement | high brightness contrast |
| Surprise | Medium | raised eyebrows | quick reaction | moderate lighting |

# S2. Generation and Selection Protocol

For each prompt, 5 independent generations were performed under identical settings. All outputs were evaluated using predefined criteria including emotional consistency, motion coherence, and visual completeness. The highest-scoring outputs were selected.
